# Cardiac Myxoma: Typical Presentation but Unusual Histology

**DOI:** 10.1155/2021/6611579

**Published:** 2021-05-04

**Authors:** Hassan H. AlAhmadi, Noor Said Alsafwani, Mohamed A. Shawarby, Fayez Ahmed

**Affiliations:** ^1^Department of Pathology, College of Medicine, King Fahd Hospital of the University, Imam Abdulrahman Bin Faisal University, P.O. Box 1982, Dammam 31441, Saudi Arabia; ^2^Department of Cardiothoracic Surgery, College of Medicine, King Fahd Hospital of the University, Imam Abdulrahman Bin Faisal University, P.O. Box 1982, Dammam 31441, Saudi Arabia

## Abstract

Cardiac myxoma, a benign heart tumor, is the most common primary tumor of the heart. Glandular differentiation within these tumors is rare, occurring in approximately 3% of all cardiac myxomas. Its presence can complicate the diagnostic process. A 43-year-old Saudi male was referred with a two-month history of progressively increasing shortness of breath. Cardiovascular examination demonstrated a soft first heart sound with a plopping sound in the mitral area and a mid-diastolic murmur. A transthoracic echocardiogram revealed a large mass attached to the interatrial septum. A diagnosis of cardiac myxoma was made, and the patient underwent en bloc resection of the mass. Microscopic evaluation of the resected mass showed a neoplastic lesion with two components: first, a typical myxoma consisting of stellate and spindle cells in a myxomatous/hemorrhagic background; second, a glandular component consisting of separate, fused, and cribriform acini embedded within the myxomatous component. The acini were lined by a single row of columnar epithelial cells with basal nuclei and apical mucin. Occasional goblet cells were also identified. The postoperative period was uneventful, and on his recent follow-up in the clinic (nine months after the surgery), the patient is doing well with no complications. Herein, we emphasize the importance of accurately diagnosing such an entity, as it can be easily confused for a metastatic adenocarcinoma, especially in patients with a history of malignancy.

## 1. Introduction

Cardiac myxoma, a benign heart tumor, is the most common primary tumor of the heart [1]. Although a common entity among cardiac neoplasms, different histological presentations may hinder the diagnostic process. Among these is the presence of glandular differentiation in rare cardiac myxomas. Ever since 1946, when Anderson and Dmytryk first described this change [2], only a few cases of “glandular cardiac myxomas” had been reported in the English literature (fewer than fifty) [3]. Herein, we report a case of left atrial myxoma with glandular differentiation, emphasizing its distinction from metastatic adenocarcinoma.

## 2. Case Presentation

A 43-year-old Saudi male was referred to the cardiology clinic with a two-month history of progressively increasing shortness of breath. On physical examination, he was anxious and relatively comfortable at rest. The chest was clear. Cardiovascular examination demonstrated a soft first heart sound with a plopping sound in the mitral area and a mid-diastolic murmur.

**Figure 1:**
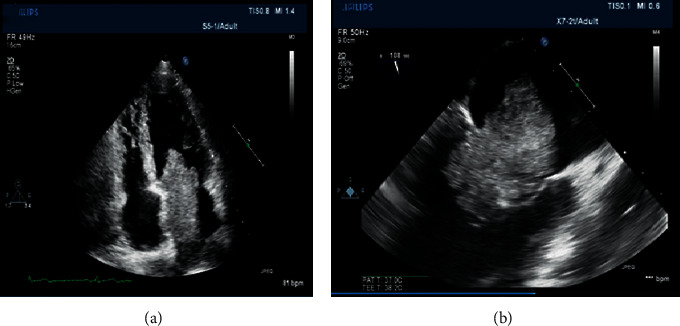
Transthoracic echocardiogram showing a large mass attached to the interatrial septum (a). The mass has a broad stalk and protrudes into the left ventricle during diastole and almost completely occludes the mitral valve (b).

A transthoracic echocardiogram revealed a large mass attached to the interatrial septum (Figure 1(a)). The mass had a broad stalk, was freely mobile, and protruded into the left ventricle during diastole with almost complete occlusion of the mitral valve (Figure 1(b)). A coronary angiogram showed no coronary artery disease.

The patient underwent en bloc resection of the mass, with wide removal of the atrial septum and patch closure with an equine pericardial patch. The anterior mitral leaflet was noticed to have an ulcerated surface. The mass was sent for histopathological examination. The operative procedure and the postoperative period were uneventful. On his recent follow-up in the clinic (nine months after the surgery), the patient is doing well with no complications.

**Figure 2:**
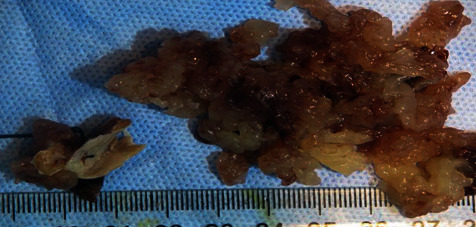
A macroscopic picture of the specimen showing the bulk of the tumor consisting of friable gelatinous tan tissue fragments with a papillary surface (on the right) along with the site of attachment (atrial septal myocardium on the left).

Macroscopically, the specimen consisted of a small fragment of the myocardium (atrial septum) and multiple fragments of gelatinous tan-white tissue, with irregular and friable surface and measuring 7.2 × 4.5 × 1.5 cm in aggregate (Figure 2).

**Figure 3:**
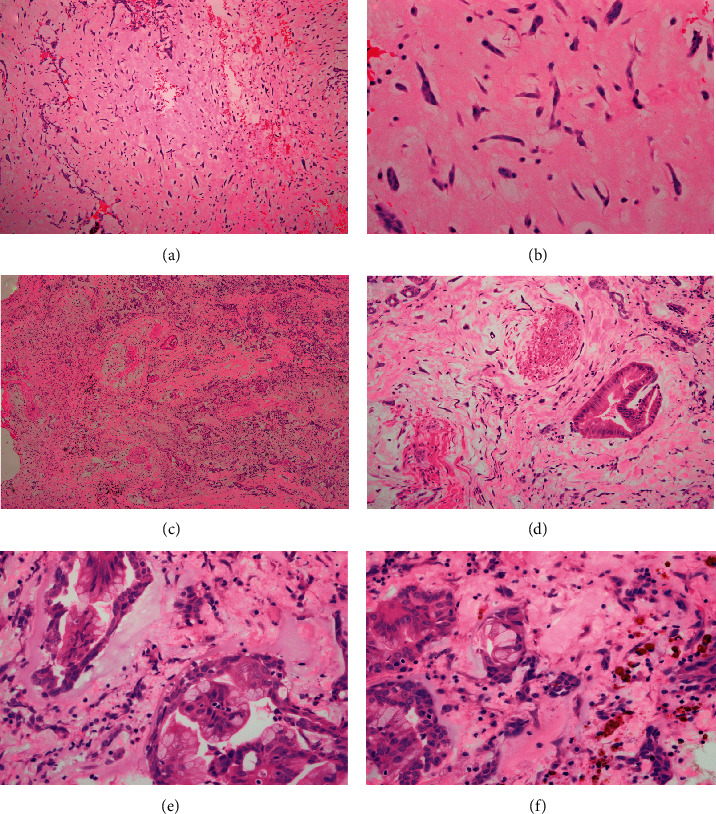
Hematoxylin and eosin stain showing the more typical cardiac myxoma cells with abundant eosinophilic cytoplasm and bland nuclei (a, b) and areas with glandular differentiation composed of bland columnar cells with basal nuclei and apical mucin (c–f). Myxoid stroma is obvious in both components, with occasional hemosiderin deposits (f) (original magnifications ×40 (c), ×100 (a), ×200 (d), and ×400 (B & E-F)).

**Figure 4:**
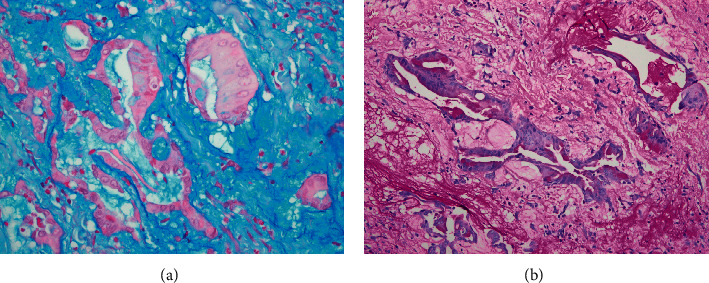
Special stains. Alcian blue (pH: 2.5) (a) and periodic acid–Schiff (PAS) (b) demonstrate mucin production within the glandular component of the tumor and the myxoid background of the tumor (original magnifications ×400 (a) and ×200 (b)).

Microscopically, hematoxylin and eosin- (H & E-) stained sections showed a neoplastic lesion with two components: first, a typical myxoma, consisting of stellate and spindle cells with eosinophilic cytoplasm, round to oval nuclei (some multinucleated), and mild nuclear polymorphism; second, a glandular component, consisting of single, fused, and cribriform acini lined by a single row of cytologically bland columnar epithelial cells with basal nuclei and apical mucin and occasional goblet cells. No significant cytologic atypia, necrosis, or stromal desmoplasia was observed. Both components were embedded in a myxomatous/hemorrhagic background with scattered hemosiderin deposits (Figures 3–4).

**Figure 5:**
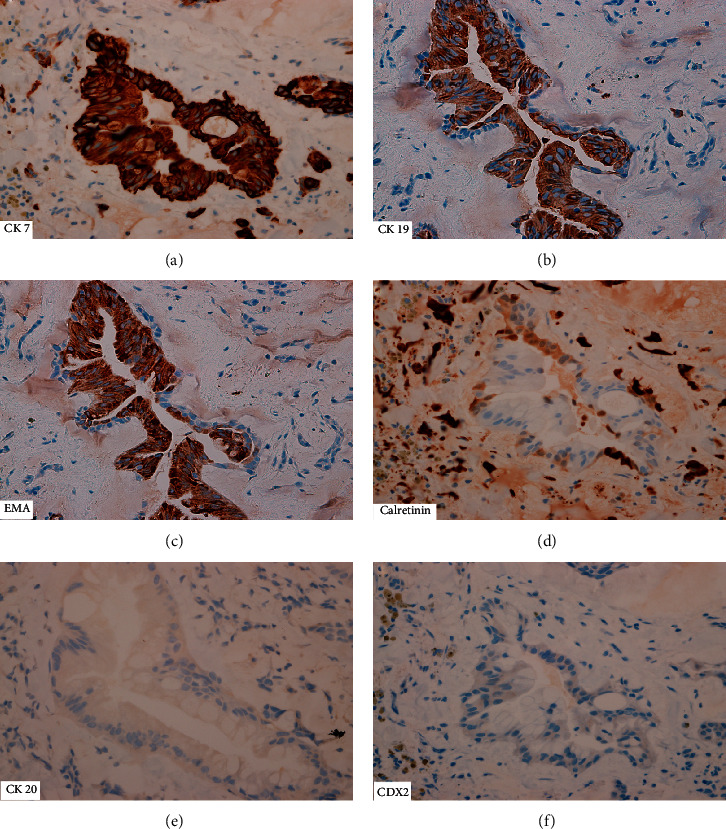
Immunohistochemical profile of the glandular epithelium showing strong immune reactivity to CK7 (a), CK19 (b), and EMA (c), while faintly and focally reactive for calretinin (d). There was no reactivity for CK20 (e) and CDX2 (f) (original magnification ×400).

Immunohistochemically, the glandular component showed diffuse reactivity for pan-CK, CK7, CK 19, epithelial membrane antigen (EMA), and carcinoembryonic antigen (CEA) and focal reactivity for calretinin (Figure 5). There was no reactivity for TTF-1, napsin, CK20, CDX2, or vimentin. Ki-67 was <10%.

Based on the abovementioned clinical, radiological, pathological, and immunohistochemical findings, a diagnosis of cardiac myxoma with glandular differentiation was made.

## 3. Discussion

Cardiac myxoma, a primary heart tumor, is the most common benign cardiac tumor [1]. It usually occurs in the atria (especially the left atrium) and presents with variable signs and symptoms ranging from an asymptomatic incidentally found lesion to shortness of breath, systemic embolization, syncope, or even death [1, 4–7]. These different presentations can be explained by the tumor hemodynamic impact dictated by its location and proximity to heart valves and its propensity to embolize as determined by the texture of the tumor (solid and smooth-surfaced vs. irregular and friable).

Under the microscope, cardiac myxomas with glandular differentiation are composed of two components: first, the typical myxoma, which includes polygonal, spindle, and stellate cells in a myxomatous background; second, acini that are lined by columnar cells with or without pseudostratification; goblets cells could also be identified [4–6]. The immunohistochemical profile of these acini in the presented case is quite intriguing. It is almost identical to the upper GIT epithelium, showing immunoreactivity for PAN-CK and CK7 while being negative for CK20 and CDX2 [3, 8].

Glandular differentiation in cardiac myxoma is well documented in the literature, with fewer than fifty reported cases [5, 9–12]. Up to our knowledge, including the presented case, forty-eight cases of glandular cardiac myxoma were reported in the English literature. Actually, glandular tissue is one of the different forms of tissues that can occur in cardiac myxomas, such as hematopoietic tissue, metaplastic bone, or thymic rests [2, 3, 5, 7]. Glandular differentiation, however, is rare in this commonly diagnosed cardiac lesion, encountered in approximately 3% of all cardiac myxomas [9]. In a large series studied by Chopra et al., out of 104 cases of cardiac myxoma, only three cases showed a glandular component [9].

Identifying glandular elements in a cardiac myxoma as an integral component of the tumor is of paramount significance due to the resemblance of such elements to gastrointestinal (GIT) epithelium, being composed of acini lined by columnar cells and goblet cells capable of producing mucin just like colonic epithelium. Not only that but also the immunohistochemistry of these acini is almost identical to GIT epithelium [3, 8]. Consequently, the presence of such glandular elements in a location outside the GIT, such as the heart, can lead to misinterpretation of the lesion as a metastatic adenocarcinoma [4].

As described in the literature [13, 14], the primary differential diagnosis included metastatic adenocarcinoma and adenocarcinoma arising within cardiac myxoma. The patient was a 43-years-old man with a history of smoking; however, several elements favor the benign nature of glandular elements in this tumor. First, no previous history of malignancy and no evidence of occult tumor was found by radiology (CT scan of chest, abdomen, and pelvis) and negative workup for other primary malignancies (for example, PSA and TTF-1 for carcinomas from prostate and lung, respectively). Second, the gross appearance of the lesion combined with the presence of typical myxoma cells in the background with the absence of necrosis, anaplastic features, mitosis, desmoplasia, and low mitotic index (Ki67 < 10%) of the glands. Third, the immunohistochemical profile is similar to the reported cases of glandular cardiac myxoma in the literature [3, 8]. All of these elements favor the benign nature of the lesion.

Indeed, occurrence in an old patient, a previous history of malignancy, a history of weight loss and anemia (which can occur due to the cardiac myxoma itself [5]), or the fact that the tumor can be fragile and embolize to different organs [7] are all factors that may blur the vision of the pathologist about the origin and nature of such a lesion and lead to an erroneous diagnosis of malignancy, especially if the patient had a previous history of adenocarcinoma [4].

Acknowledging the difficulty of differentiating glandular elements in cardiac myxoma from metastasis as outlined above, with careful gross and microscopic examination of the lesion, with thorough clinical and radiological workup to exclude malignancy elsewhere, can help to distinguish it from malignancy. The soft, gelatinous gross appearance, along with the absence of significant nuclear atypia, necrosis, mitosis, and desmoplasia, along with the presence of the typical myxoma component, all stand against the diagnosis of metastatic adenocarcinoma.

## 4. Conclusion

We report a case of a 43-year-old male diagnosed with cardiac myxoma with glandular differentiation. Up to our knowledge, this is the first case to be reported from Saudi Arabia. Due to the rarity of such a lesion, we emphasize to the pathologist the importance of accurately diagnosing this entity, which can be confused for metastatic adenocarcinoma, especially in patients with a history of malignancy.

## Data Availability

All the data utilized in the case report are available from the corresponding author upon request.

## Abbreviations

CDX2: Caudal-type homeobox 2
CEA: Carcinoembryonic antigen
CK 7: Cytokeratin 7
CK 5/6: Cytokeratin 5/6
CK 19: Cytokeratin 19
CK 20: Cytokeratin 20
CT: Computerized tomography
EMA: Epithelial membrane antigen
GIT: Gastrointestinal
H&E: Hematoxylin and eosin
PAS: Periodic acid–Schiff
PSA: Prostate-specific antigen
TTF-1: Thyroid transcription factor 1.
